# Why is pet goods consumption imperceptible for economists? A scoping review

**DOI:** 10.1007/s43546-022-00349-5

**Published:** 2022-10-13

**Authors:** N. Gromek, J. Perek-Białas

**Affiliations:** 1 Warsaw School of Economics, Collegium of Economic Analysis, Institute of Statistics and Demography, Madalińskiego 6/8, 02-513 Warsaw, Poland; 2 Jagiellonian University, Institute of Sociology, ul. Grodzka 52, 31-044 Cracow and Centre for Evaluation and Analysis of Public Policies, Gołębia 24, 31-007 Cracow, Poland

**Keywords:** Pets, Pet goods consumption, Expenses on pet goods, Scoping review

## Abstract

Nowadays, pets more frequently are becoming family members which deserve certain products and goods, as well as services. In this way, pets are becoming consumers even they do not have a possibility to make decisions (as opposed to human being) as we analyze taking into account human being. Recently pet-related topics are gaining more attention in the press and among researchers in the field of marketing and psychology. Numerous articles regarding pet-related business patterns, like pet insurance, day care and pet friendly hotels are published. No wonder, the popularity of pets among households has been growing for many years. In this article, a scoping review aimed at identifying available studies about expenditures on pet goods and owners’ economic consumption choices has been conducted. A comprehensive search strategy was used across Scopus and EBSCO database. The results show that there is only a few studies concerning pet goods consumption through the lens of economic theories. As such this topic in not explored enough while the market of goods and services is growing.

## Introduction

These days, about 67% of American households own a pet. The number of US households with pets increased from 66.50 million to 84.90 million between 2012 and 2020, and the this upward trend will continue (APPA 2021).

Many pet owners feel not like a pet owner but like a pet parent. In this way, the phenomenon of pet parent shows that people do not regret spending money on their ‘babies’. It’s no wonder that 83% of pet owners identify to themselves as "Mommy" or "Daddy” (Morais 2004).

Pets become new members of the family and should be considered in the economics of household in the same way that other members of the family are. Household animals thrive in today’s culture due to contemporary lifestyles such as maternity leave, flexible working hours, and later marriage. Furthermore, COVID-19 is expected to increase the popularity of owning pets (Oliva and Johnston 2021; Giansanti et al. 2022).

While the economics aspects of the family/household might stretch out to pets, as confirmed by regular references to pets as "child" there are various differentiations among pets and kids: it is legitimate to purchase pets, yet not kids; undesirable pets can be deserted; and pets cannot accommodate guardians in their advanced age. Single people can possess pets without the shame that accompanies being a solitary parent. Pets have additionally stood out enough to be noticed in legal strategies until of late.

**Fig. 1:**
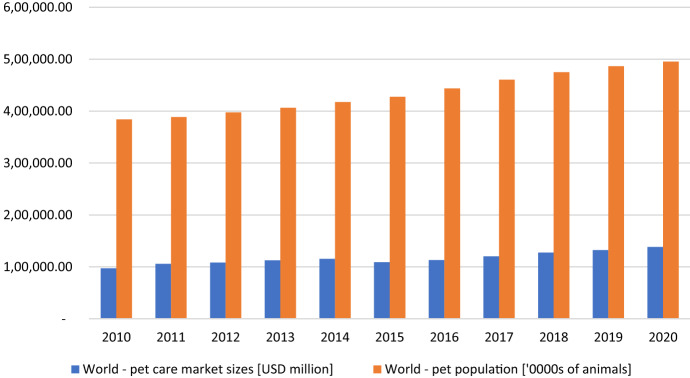
Global pet goods market value and population of pets. Source: *Global Marketing Information Data Base (GMID) Euromonitor International,* available at: https://www.portal.euromonitor.com/portal/magazine/homemain, access: [17.02.2021]

The worldwide pet goods market value has been growing systematically for last 10 years, reaching the value of USD 138.24 million (including the value of the pet food market—USD 98.07 million and the value of pet products—USD 40.18 million) in 2020 (GMID 2022) (Chart 1). Here, the pet goods market includes of: the pet food market, the cat litter market, the veterinary services market, the pet supplements market, and the market for other pet products. This value increased by 42% compared to 2010 (including the value of the pet food market by 38% and the value of the pet products market by 52%) (GMID 2020). The number of pets in the world has also been increasing systematically since 2010. Over the past 10 years, the population of domestic animals, i.e., cats, dogs, aquarium fishes, domestic birds, small mammals and domestic reptiles, has increased by 29% in 2020 and amounted to 4,952,320,000.72 (GMID 2022). Just in the UK, in just one year, an average, weekly households' expenditure on pet goods consumption household rose by 675% from £ 0.80 to £ 5.40 (Office of National Statistics 2017). While in US, expenditures connected with pets amounted up to 1% of total households’ expenditures and were higher than average expenditures on alcohol, stationary phone payment and men and children clothes and an average yearly expenditures of households on pet food were higher than average expenditures on sweets, bread and poultry (Henderson 2013) (Fig. 1).

As such, this is no longer a service niche, dominated only by small aquarium shops as it used to be 20 years ago (Frątczak-Rudnicka 2015; GMID 2022). Nowadays, this market consists of: veterinary clinics, toys and accessories (including electronic gadgets), pharmaceuticals, clothes, professional literature, TV channels and internet portals for pets, and even cemeteries for animals. The more sophisticated market of pet services is also gaining importance: cosmetic, hairdressing, dietary or hotel services (Frątczak-Rudnicka 2015). Taking into account that this market is growing it has to be noticed that the economic literature has to be somehow recognized the way how the pet consumption is described, analyzed and as well developed with conclusions which can contribute to the research and also implications for this business.

The paper’s aim is to present the results of scope review in which the main question asked was: in what way and what new findings could be found in the literature when we would like to analyze the pet consumption especially expenses on pet goods and pet owner economic choices? Shortly, the aim was to see if and how the link to the economic literature of this consumption is presented in available publications. Two detailed research questions were added:

- Are there any studies that analyze pet goods consumption through the lens of economic theories?
- And which economic theories are included in studies concerning pet goods consumption and how they are presented?

In this paper, after a short theoretical context, methodology of scope review was detailed described with sum up of the results. The paper ends with conclusions and limitations.

## Theoretical background

A wide range of studies related to pet goods consumption can be already found in the international literature, but those pieces are primally focused on consumer side (Archer 1997). There also researches who were focused on people’s motives of owning pet (Zasloff and Kidd 1994), relations between pet and pet owner (Ellson 2008) and influence of pet owning on pet owners’ health e.g., blood pressure (Karen 2003).

According to other studies pet owners consider pet as a family member, they frequently let their pet sleep in the bed with them, their pets get presents and they participate in their holiday (The Harris Poll 2012). There are even researches suggesting that pet owners treat their pets like a family member or even as a child—term known as a ‘fur baby’ in the American literature (Greenebaum 2004). There are many economic well-known theories and studies that cover consumer behavior and economic decisions among families with children—e. g. Gary Becker’s (Becker 1976) and Harvey Leibenstein’s considerations (Leibenstein 1957). On the other hand, there are not economic theories and studies related to consumer behavior and economic decisions among families with pets. There are many unanswered economic questions connected with pet ownership: *Are there economic reasons why people own pets?, Do pets have an influence on family members utility?, Can we notice the substitution effect among families with pets (where goods are pet goods and leisure services)?*

In the neoclassical theory of consumer choice people make their consumption choices to maximalise utility (described by preferences) subject to an individual budget constraint. It shows how the consumers should choose if they make rational choices and use utility maximizing model (Varian 2019). For various types of goods, income and price elasticity of demand is analyzed, as well as income and substitution effects of the price changes are studied. The diminishing marginal utility is also assumed. Extensions of the basic model show how the decisions are made in a household and could be studied for different types of households.

Pet goods consumption has not been included in considerations in following economic consumption thesis as: absolute income hypothesis (Keynes 1936), relative income theory (Duesenberry 1949), permanent income hypothesis (Friedman 1957), life-cycle theory of consumption (Ando and Modigliani 1963) and conspicuous consumption (Veblen 1899). In other words, there are not economic studies that detailed explain:

- If households’ consumption on pet goods function is an economic formula that represents the functional relationship between total households’ consumption of pet goods and households’ income.
- If the households’ pet goods consumption decisions are motivated by "relative" consumption concerns.
- If households’ level of pet goods consumption depends not only on its current income but also, on its long-term expected earnings.
- If households’ preferences regarding pet goods are determined to the position of each individual in the social hierarchy.

## Materials and methods

### Searching strategy

A comprehensive search string on ‘pet goods consumption’ was developed for searches in two typical for social science electronic databases—Scopus and EBSCO (Academic Research Source eJournals [EBSCO] up until March 2021). Titles first and then abstracts were checked for inclusion by two raters (NG and JPB) after an initial step of deleting duplicates and irrelevant entries. Records were assigned to reviewers at random, and conflicts were settled by consensus with a joint agreed decision about the publication.

For each of the articles included in the original search, a snowball search was undertaken to find additional records for full-text inspection using Google Scholar's "related to" and "cited by" tools (Atkinson and Cipriani 2018). Additional papers based on specialist expertise on the topic of pet product use have also been added. Additional articles were found by looking through the bibliographies of the final batch of records. At the final stage of the procedure, two raters independently reviewed the full text (JPB, NG).

Eligible studies met the following inclusion criteria: (a) only published articles, (b) published in English between 2000 and 2021 (03.31.2021), (c) documents where one world from the following list have appeared in title or abstract or keyword: ("pet owner", "per related", "domestic pet", "home pet", "pet studies", "pet consumption", "pet co-consumption", "keeping pet", "spending on pet", "pet spending", "pet care cost", "pet ownership cost” and (d) documents where one world from the following list have appeared in keyword: "consumption", "expenditures", "outgoing", "outlay", "expenses", "outgoes".

The following exclusion criteria were agreed to take into account: (a) other types of document: Conference Paper, book chapter, conference Review and review, (b) documents where one world connected to Polyethylene terephthalate (PET) from the following list have appeared in title or abstract or keyword: “polyethylene”, “petrol”, “Physiologically equivalent temperature”, “plastic”, “polymeric”, “methyl” and (c) documents where one world connected to Positron emission tomography (PET) from the following list have appeared in title or abstract or keyword: “AAFP”, “AAHA” OR “serotonine*” OR “tomography*” OR “SPECT*” OR “CNR*” OR “fMRI*” OR “tDCS*” OR “DBS*” OR “molecular*”).

### Publications selected for review

**Fig. 2:**
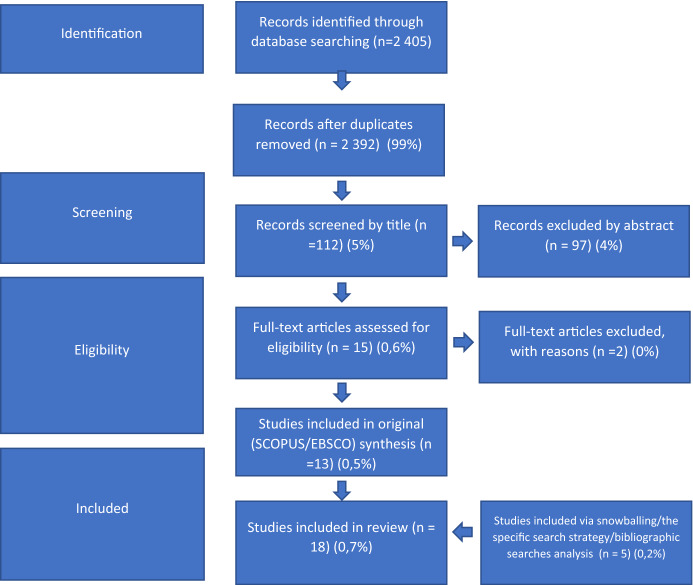
Prisma diagram

A total of 2405 records were retrieved based on the original search. There were 13 duplicates. 2392 records have been screened by title and most of the articles were excluded because: they were related to medical research ("PET" as Positron emission *tomography*, despite the exclusions in the keywords, there were many articles), they were related to environmental, chemical research ("PET" as *Polyethylene terephthalate*, despite the exclusions in the keywords, there were numerous articles). Based on the title screening 112 articles were identified for abstract review. Based on abstract review 97 of them have been excluded, so 15 articles found in databases were identified for full-text review. The remaining 97 articles dealt with the possession of pets in a household in a different context, not including pet goods consumption. The most common subject related to keeping a pet is the analysis of its impact on the health of the owners, mainly on the risk of asthma. Other popular topics related to pet keeping are: analysis of the mental support/well-being of a pet owner related to owning a pet, an analysis of the emotional relationship between a pet owner and a pet, an analysis of people's attitudes toward pets, and an analysis of pet owners' problems with renting an apartment. After full-text review 2 articles have been rejected—one because language (article in German) (Pütz and Poerting 2020), second because of very specific topic—prescribed consumption in case of pet goods consumption (Lamour and De La Robertie 2016). In the end, 13 articles based on the original research have been emerged. Additional 12 records were identified as potential important based on the bibliographic check of selected papers but based on abstract review 7 of them have been further excluded. Finally, this resulted in 18 records included in this scope review (See Fig. 2). Details on the characteristics of the different studies included in the review are provided in Table 1.

**Table 1:** Articles by research type, sample description and size, research technique

References	Research type: quantitative or qualitative, mix-mode or theoretical paper	Research technique	Sample description	Sample size
Maharaj et al. 2018	Theoretical paper	Literature review	Studies that cover pet-oriented leisure and pet goods consumption topics Author divided studies into 3 most important sections of pet-oriented leisure and consumption: 1.Trends in leisure and consumption 2.Psychological perspectives 3.Implications of pet ownership Analyzed country: Studies from many countries e.g., Australia, United States	About 185 studies
Vänskä 2014	Theoretical paper	Literature review and own memories	Memories/experiences: 1.First author memory—Jack London’s (1903) The Call of the Wild 2.Vital childhood memories when the author has been strolling the corridors of a shopping mall Articles about relationship between humans and dogs Analyzed country: Many countries but not mentioned particular	2 memories and 27 studies
Vänskä 2016	Theoretical paper	Literature review	Literature consist of following areas: 1.Posthumanist theoretical framework 2.Cultural history of the pet dog treated like a source and mediator of positive emotions 3.Emotional bond between dogs and humans Analyzed country: Many countries but not mentioned particular	About 60 studies
Syrjälä 2016	Theoretical paper	Literature review and analytic autoethnography with a plentitude of supplementary data	Literature that addresses transformative processes in more general terms in consumption Analyzed country: Many countries but not mentioned particular	About 110 studies
Chaumet et al. 2021	Quantitative	Survey	Pet owners at the primary investigator’s hospital Analyzed country: United States	235 pet owners
Chen et al. 2012	Quantitative	Survey	Quota sampling was used to recruited 685 dog owners. A total of 578 valid responses were included in the survey sample, resulting in an 84.3% valid return rate Analyzed country: Taiwan	578 pet owners
Tesfom and Birch 2010	Quantitative	Survey	The information was gathered through a survey of dog owners at two Washington higher education institutions: Eastern Washington University and Bellevue Community College. A total of 1300 dog owners were surveyed Analyzed country: United States	1300 pet owners
Koppel et al. 2018	Quantitative	Survey	The screening criteria were used to select participants. Only Thai consumers over the age of 18 who had dogs in their homes, fed their dogs dry dog food, and decided or helped determine what dog food to buy were recruited for the research Analyzed country: Taiwan	120 pet owners
Williams et al. 2020	Quantitative	Survey	A national survey of dog owners with and without pet health insurance Respondents were chosen at random by Qualtrics' panels and are indicative of the general population in the United States Analyzed country: United States	654 dog owners
Gates et al. 2019	Quantitative	Survey	Respondents over the age of 18 Analyzed country: New Zeland	1572 pet and non-pet owners
Kirk 2019	Quantitative	Survey (first and third study), experimental design (second study)	99 university employee that own dog or cat owners participated in the primary experiment. In the second one, 200 Mturk employee that own a pet. In the third one 120 Mturk workers that own a pet Analyzed country: Not mentioned	419 pet owners
Schwarz et al. 2007	Quantitative	Secondary data (Consumer Expenditure Survey)	Twenty years of data from the Consumer Expenditure Survey Analyzed country: United States	113,380 households
Wolf et al. 2008	Quantitative	Secondary data	US consumers from 1980 through 2005 Analyzed country: United States	Approximately 5,000 households completing the survey each quarter from 1980 through 1998 and 7,500 households completing the survey beginning in 1999
Jyrinki and Leipamaa-Leskinen 2005	Quantitative	Survey	Women were overrepresented in the sample in terms of general representativeness, but the demographic and socioeconomic profiles of the respondents were in line with the general features of the Finnish population Analyzed country: Finland	264 pet owners
Kylkilahti et al. 2016	Qualitative	Focus groups	Interviewees that have been recruited through snowball sampling and during fieldwork in pet supply stores, vet clinics and communal dog parks. In the sample, there were different kinds of pet owners: pet hobbyists, and pet owners who are not involved in any particular hobbies with their pets Analyzed country: Finland	40 pet owners
Mosteller 2008	Qualitative	In-depth interviews	Six informants were interviewed in depth, and the researcher chose them based on firsthand knowledge of their pet history and animal experience. The informants ranged in age, education, income, the existence of children, and marital status Analyzed country: United States	6 pet owners
Brockman et al. 2008	Qualitative	In-depth interviews	In-depth interviews were conducted with informants who had previously made a decision concerning costly veterinary treatment. Informants were found through acquaintances of the writers' friends or a professor and staff e-mail list at the university Analyzed country: United States	13 pet owners
Ridgway et al. 2008	Mix method	First study Qualitative and second study Quantitative (survey)	1.First study: Women with pets. Respondents who are shopaholics and spend a lot of money on their pet 2.Second study: Customers of a clothing business via the internet—with and without pets Analyzed country: United States	1303 pet and non-pet owners (1294 pet/non pet owners in quantitative study, 9 pet owners in qualitative study)

It is worth highlighting that most studies were conducted in English speaking, high-income countries, including Scandinavian countries, Taiwan and the United States of America, and that no studies were conducted in low and lower middle income countries. A brief sum up of the research selected for the analysis is presented in Table 2.

**Table 2:** Articles by purpose, results and results in the context of pet goods consumption

References	Purpose of the study	Results of the study	Results in the context of pet goods consumption
Maharaj et al. 2018	To explore pet-oriented leisure and consumption via psychological lens	Pets have a positive impact on people's self-esteem and happiness through offering pleasurable recreational activities. There is little evidence of the influence of pet-related expenses on health	**Pets have transformed from consumers beings into co-consumers.** To highlight that pets are the common consumers, author describes services to meet the travel needs of pets and their owners including airlines, hotels and dog hospitality services
Vänskä 2014	The theoretical investigation of the “*babyfied*” dog and the turbulent relationship between dogs and parenting in contemporary consumer culture	Real animals, especially small dogs, have begun to replace teddy bears and other stuffed animals as the dressed-up childlike animal, in tandem with the new educational attitude toward pets and animals in general	**Consumerism aimed at dogs demonstrates how alive and well-functioning modern capitalism is.** Media coverage of fashions to dogs underlines that dogs work as marketers, ambassadors and distributors of high fashion and luxurious consumption
Vänskä 2016	To investigate how pet dog goods identify and materialize the ideal emotional connection between a person and a dog	Dog fashion shows how the desire to create a bond between a person and a dog is translated into tangible products and services	**Emotional bond between pets and their owner is very important in case on pet goods consumption.** Traditional humanist ideas about families, parenting, and childhood are challenged by the dog
Syrjälä 2016	To portray the defining moment of the change that happens when a casual enthusiast turns into a serious hobbyist inside the subculture of dog agility devotees	Enthusiast at the turning point becomes a serious hobbyist who engages with a multitude of dog-related businesses to establish his or her seriousness	**There are four different nature and manifestation of dog-related consumption.** These positions empower catching the manners by which the changes at the defining moment are supported, differentiated and created corresponding to various social assets and settings
Chaumet et al. 2021	To investigate the perception and frequency of veterinary insurance among owners in a specialized small animal hospital around a large metropolitan city in the US	28.5% owners reported having at least one pet insurance, most of them—77.6% reported that they are satisfied with their insurance plan and would recommend their current insurance plan to a friend—73.2%. Most of the pet owners choose their current insurance plan based on internet research (40.3%)	**In the article there is a description of spending on pet goods (pet insurance)—amounts, percentage of owners that buy goods**
Chen et al. 2012	To examine how owners' consumption values and behavioral habits may be segmented and promoted strategically based on their human-pet relationships	This study established three segments of pet owners using pet-related services. The first cluster consisted of *Anthropomorphic* owners who place a high value on efficiency, the second of *Attached* owners who search, and the third of *Owners* who seek engagement and the economic value of a service	**The type of relationship that exists between pet owners and their pets can be used to create consumption value and behavior clusters.** This study found that human-pet relationships have a variety of effects and that pet owners vary in terms of consumption beliefs, knowledge search habits, and retail selection preferences, using a variety of pet services
Tesfom and Birch 2010	To determine whether dog owners buy for their dogs the way they buy for themselves	Dog owners are more loyal to dog food brands than to human food brands, according to the report. Dog owners have often found to be more open to the price of human food than to the price of dog food	**There is a correlation between spending on pets good consumption and spending on owners' goods.** Dog owners have also been discovered to be more open to their own food costs than to the prices of their dog's food. This may be because dog food accounts for a small portion of total spending, so paying a high price for it has no effect on overall spending
Koppel et al. 2018	To determine what aspects of pet food appearance characteristics determine higher liking among pet owners	Several consumer clusters have been discovered, indicating that consumer preferences differed when it came to such characteristics such as kibble shapes and colors	**There are products' characteristics that may have an influence on owner's purchase decisions.** Several factors influence the purchase decision of pet goods e.g.: product price, packaging, brand etc
Williams et al. 2020	To measure which dog and dog owner characteristics may have the impact on the frequency of veterinary visits and expenditures on veterinary pet healthcare	Pet health insurance had a significant and positive impact on the amount spent on the veterinarian. Having pet health insurance raise the amount spent at the visit while having pet health insurance had no effect on the frequency of visits	**There are some owners' characteristics that have an influence on expenses on pet goods.** Expenses on pet care are statistically significantly correlated with: owner’s income, having insurance, other expenses on pet goods, past pet’s incidence of diseases and expected future expenses on pet care
Gates et al. 2019	To describe the demographics of and predictors for pet ownership, reasons for visiting a veterinarian, and pet-related expenditure among pet owners in New Zealand	The most popular purpose for owning dogs, cats, and birds is companionship. Pet ownership is more likely among respondents who live in a rural area, have a higher household income, with children, and female. Pets’ health problems is the most popular factor to take it them vet	**There are some owners' characteristics that have an influence on willingness to go to veterinary.** Owners who treat their pets as friends, with higher income, and owned dogs or cats rather than other animals were the most likely to have taken their pet to the veterinarian
Kirk 2019	To check if the feelings of ownership, or psychological ownership, play consumers' economic valuation of their pets	Customers place a higher economic valuation on dogs versus cats, as evidenced by willingness to pay greater for pet goods, as well as increased word-of-mouth about the pet. This impact is defined through customers superior mental possession of and ensuing emotional attachment to the pet	**Owner's emotional attachment to their pets has an influence on spending on pet goods.** Research has demonstrated that consumers place a higher economic valuation on dogs than cats
Schwarz et al. 2007	To investigate the applicability of the economics of the household to the pet ownership decision and pet expenditures	Pet ownership and expenditures are lower in households with very young children, indicating a substitution link. Pet ownership is more common in households with older children, implying a complimentary relationship. Pet spending is lower in households with additional children, indicating a substitution relationship	**There are some households' characteristics that have an influence on expenses on pet goods.** In married homes, having more children reduces pet spending, implying that children and pets tend to be substitutes
Wolf et al. 2008	To evaluate US consumer expenditures for veterinary services, pets-pet supplies, and pet-related services	Total expenditures on pet-related and veterinary services increased from 1980 to 2005, as did the percentage of families with such an expenditure. The percentage of households that spent money on veterinary services remained relatively steady	**There are some households' characteristics that have an influence on expenses on pet goods.** The probability for pet-related and veterinary service expenditures increase with income, education, and family size and is higher for household heads who are white, married, owned their residence, and lived in a rural area
Jyrinki and Leipamaa-Leskinen 2005	To explore how consumers’ seeing their pets as extended self can be found to explicate their pet food consumption	The construct of extended self-relating to pets consists of three opposing aspect pairs: personal and social, symbolic and functional as well as attachment and control. Those consumers who regarded their pets as their self-extensions differ from other respondents and consume pet food differently than others	**There is a connection between seeing pets as extended self and consumption behavior in pet food.** The concept of extended self does play a role in consumer behavior when it comes to pets
Kylkilahti et al. 2016	To develop a conceptual understanding of co-consumption by examining how pets act as co-consumers in everyday consumption	The pet owner and the pet have consuming experiences in which they engage with other actors like service providers. Pet owner consumes because of the pet and must continuously consider pet in decisions and activities other than pet consumption, such as what sort of car to buy, where to work, who to marry, and so on	**Pets appear in three roles as co-consumers.** Pets as co-consumers of products, services and experiences participate in consumers’ everyday life in three ways: (1) consumption because of the pet, (2) consumption for the pet and (3) consumption of the services provided by the pet
Mosteller 2008	To explore the meanings and roles pets play in peoples' lives	Relationship theory helps explain the drivers behind pet-acquisition growth. A consumer – pet relationship framework is used to identify factors that may influence the strength and duration of consumer – pet relationships	**Consumers who perceive their pets to reinforce their self-concepts, elevate their social status, and be integrated members of family or social networks are associated with positive pet–human relationships**. The physical proximity between the pet and consumer may positively reflect the consumer's emotional attachment to the pet
Brockman et al. 2008	To examine the decision-making process consumers go through when facing expensive medical treatment for their pets	Different types of emotional attachment were discovered and tend to correlate with the amount and likelihood of pursuing treatment, similar to previous consumer-behavior research	**Consumers' emotional relationship to animals has a significant impact on the nature of their veterinary care decisions.** Consumers with moderate and low attachments make cognitive and reasoned decisions. Consumers who regard their dogs as cherished others and elevate them to family-member status, make more emotional rather than logical pet-health decisions
Ridgway et al. 2008	To examine how a predisposition to buy excessively for one's self connects to spending on one's pet using two research	Respondents who score high on an excessive buying index also tend to spend more on their pets	**There is a relationship between a tendency for excessive buying and spending for one's pet.** One reason for this relationship seems to be that the participants form very strong attachments to their pets and consider them to be their children

## Results

The vast majority of articles concerning pets in households refer to their impact on the health of their owners (mainly on asthma) (i.e., Medjo et al. 2013; Shirai et al. 2005). The other researches (i.e., Kim et al. 2020; d’Ovidio and Pirrone 2018) are focused on people’s motives of owning pet and relations between pet and pet owner. Other very popular topics concerning keeping pets in households: the mental support / well-being of a pet owner related to owning a pet and an analysis of pet owners' problems with renting an apartment (i.e., Power 2017; Chee 2017). Only a few articles concerning pet goods consumption but definitely most of them is on the marketing field. The percentages of studies grouped by the main topics have been shown in Table 3.

**Table 3:** Articles by main topics

Articles by main topics	Percentage of studies (*n* = 18)
Pets as family members	16,67%
Human motives I relation to pets	27,78%
The welfare of the pet’s owner	5,56%
The consumption of pet goods	11,11%
The relationship of children with pets	0,00%
The family view to pets	5,56%
Family spending on pets	11,11%
The tendency to buy pets	–
Spending on fashion and accessories for pets	5,56%
Pets insurance	11,11%
Pets trademarks	5,56%

Source: own preparation

Human-animal bond is the most popular variable in literature that has correlation with expenditures on pet goods consumption. The human-animal bond is not just between people and traditional companion animals, such as dogs and cats. People develop relationships with birds, pocket pets, reptiles, and large animals, including food production animals. Human-pet bond is the most common variable in the literature that may have an influence on pet goods consumption (Vänskä 2016; Kirk 2019; Gates et al. 2019; Chen et al. 2012). Many people believe their pets are their soulmates. Strong emotional bond between a pet owner and a pet (pet parent, co-consumption phenomena) meaning that people do not regret spending money on ‘babies’. Pets live with people and participate in people's everyday life activities and they are often seen as human‐like family members. Consumers from the industrialized countries are investing more money in their pets and spending more time with them than ever before. The pet with its owner can be even considered as a form of an unit that consumes together (Kylkilahti et al. 2016; Maharaj et al. 2018). Ridgway et al. (2008) proved a relationship between a tendency for excessive buying and spending on their's pet. This relationship seems to result of very strong attachments to their pets and considering them to be their children. They report feelings of being better “parents” if they take care of their pets by spending excessively on them. Respondents who score high on an excessive buying index also tend to spend more on their pets for toys, food treats, clothing, accessories, and grooming products. According to Vänskä (2014) the anthropomorphized animal plays a crucial role in building and comprehending the romantic ideal of childhood and innocence. Real animals, especially small dogs, have begun to replace teddy bears and other stuffed animals as the dressed-up childlike animal, in tandem with the new educational attitude toward pets and animals in general. The same author highlighted that pet dog and human are built as a team that consumes together and has shared user experiences. Pet dog fashions show how the desire to create a bond between a person and a dog is translated into tangible products and services (Vänskä 2014). Mosteller (2008) explored the meanings and roles pets play in peoples' lives. Relationship theory helps explain the drivers behind pet-acquisition growth. According to him consumers who perceive their pets to reinforce their self-concepts, elevate their social status, and be integrated members of family or social networks are associated with positive pet–human relationships. Brockman et al. (2008) shown that consumers' emotional relationship to animals has a significant impact on the nature of their veterinary care decisions. Consumers with moderate and low attachments make cognitive and reasoned decisions. Consumers who regard their dogs as cherished others and elevate them to family-member status, even spiritual being status, make more emotional rather than logical pet-health decisions. According to Chen et al. (2012) the type of relationship that exists between pet owners and their pets can be used to create consumption value and behavior clusters. This study found that human-pet relationships have a variety of effects and that pet owners vary in terms of consumption beliefs, knowledge search habits, and retail selection preferences, using a variety of pet services.

Although the most frequently cited variable influencing the pet goods consumption is human-animal bond, there are also observable variables that can determine it. Other variables that is connected with expenses on pet goods is household’s income. According to Williams et al. (2020) the amount households spent on pet care rose with income. Owners with an income of more than $55,000 spent an average of $164 more than those with a household income of less than $55,000. Pet owners with a household income of more than $55,000 may be more willing/able to spend more on their pets and pet-related expenses since their disposable income permits them to spend beyond essential living needs like rent, food, and petrol. Expenses on pet care are also statistically significantly correlated with: having insurance, other expenses on pet goods (e.g., toys, feed, etc.), past pet’s incidence of diseases and expected future expenses on pet care. Higher income respondents were more likely to say they had taken their pet to the veterinarian, particularly if the visit included costly emergency treatment (Gates et al. 2019). Owners with a higher income preferred more regular, more costly vet care, implying that those with more disposable income were more likely to invest in their dog's health and well-being. The probability for pet-related and veterinary service expenditures increased with income, education, and family size and was higher for household heads who were white, were married, owned their residence, and lived in a rural area (Wolf et al. 2008). According to Schwarz et al. (2007) pet expenditures are lower in households with very young children, indicating a substitution link. Pet spending is lower in households with additional children, indicating a substitution relationship. The effect of income on pet ownership and pet spending is combined in the income elasticities computation, women in married families had lower income elasticities for pet expenditures than males. As has been shown most researches who explore observable variables that determine expenses on pet goods analyze veterinary/pet insurance market. Those expenses are familiar for pet owners—28.5% owners reported having at least one pet insurance (Chaumet et al. 2021).

A wide range of studies related to pets goods consumption are primarly focused on consumer side (Koppel et al. 2018; Tesfom and Birch 2010; Jyrinki and Leipamaa-Leskinen 2005). Koppel et al. (2018) demonstrated that there are products' characteristics that may have an influence on owner's purchase decisions. Several factors influence the purchase decision of pet goods (in that article dry dog food), such as product price, packaging, brand, marketing claims, product extrinsic characteristics (aroma, appearance, color, shape, size), whether the companion animal will consume the food, and if they do, what are the digestive and health consequences. Tesfom and Birch (2010) highlighted that there is a correlation between spending on pets good consumption and spending on owners' goods. Moreover they indicated that dog owners are more loyal to dog food brands than to human food brands and dog owners have often found to be more open to the price of human food than to the price of dog food. What is more, dog owners are more committed to purchasing healthy dog food than they are to purchasing healthy human food. According to Jyrinki and Leipamaa-Leskinen (2005) there is a connection between seeing pets as extended self and consumption behavior in pet food. The concept of extended self does play a role in consumer behavior when it comes to pets. Consumers who see their dogs as extensions of themselves vary from other respondents in case of pet food consumption. Among the extended self group, price and quality awareness, pleasure providing, self-esteem, knowledge, and pet feeding planning were highlighted. Syrjälä (2016) depicted the turning point in a casual enthusiast's transformation into a serious hobbyist inside the community of dog agility devotees. According to her the enthusiast at the turning point becomes a serious hobbyist who engages with a multitude of dog-related businesses to establish his or her seriousness.

## Discussion

Pet goods consumption is not the main area of interest to researchers so far. For this reason, conducting a literature review in this field was a meaningful challenge and it was confirmed by limited number of papers found and selected for this review. A reflection of the difficulties in conducting this type of analysis is, has been for example, adding as many as five articles from outside the databases (Scopus and EBSCO), which constitute almost 1/3 of all articles included in the review. The analysis is of course limited to Scopus and EBSCO but on the other hand all relevant work should be found as rather including other often used databases like: PsycINFO, ProQuest Central, SAGE Premier and Science Direct. The analysis of pet goods consumption are focused mainly on marketing field. In particular they deal with categories such as preferences, segmentation, consumer behavior which are mostly recognized to and appraised by marketing field. Researchers that study pet goods consumption do not focus on fundamental for economists topics: demand elasticities, testing the law of diminishing marginal utility, microeconomic utility etc.

Even the scope review confirmed that some of the papers accord with well-known microeconomic theories mentioned in theoretical background like absolute income hypothesis or life-cycle theory of consumption or conspicuous consumption, still there are gaps. As reference to absolute income hypothesis (J.M. Keynes) could be found in Wolf et al. 2008; Williams et al. 2020; Gates et al. 2019 but those studies have not been analyzed via economic theory lens and Tesfom and Birch 2010—this study was only linked to marketing field. In case of life-cycle theory of consumption (F. Modigliani, A.K. Ando)—here Williams et al. (2020) indicated that expenses on pet care are statistically significantly correlated with expected future expenses on pet care, but the life-cycle theory of consumption theory in case of pet goods consumption has not been analyzed in economic way in their work. And last conspicuous consumption (T.B. Veblen) found in Syrjälä (2016) shown that some dog owners manifest their pet goods consumption that going to extreme lengths in consumption and spending but there was not used economic approach. Also there are some theoretical papers like Maharaj et al. 2018; Vänskä 2014, 2016 and Syrjälä 2016 but the aim of those literature reviews were completely different. Authors put leisure activities with pets, emotional bond between pets and owners, and transformative processes of pet consumption over expenditures on pet goods or owners’ economic consumption choices.

Based on the scoping review, it is impossible to create an economic model to express the consumption function for pets and so far it was not found (via scoping review) adequate economic literature that shows the main components of consumption function for pets (owners’ income and owners’ expenses). Moreover, it was impossible to show a demand/supply curves for pets because we could not control e.g., how many pets households have, as well the pets’ price. Finally, we did not find papers which show analyses the influence of pet on consumers’ real income.

This scoping review indicated the lack of economic studies which look at the pet consumption via lens of the economic consumption theory and modeling approach. As stated in the introduction pet goods market has been growing steadily for many years and is the prospective one. So, our analysis confirmed that economists are not interesting in more advanced analysis of pet consumption, while pets have been gaining attention among researchers in the field of marketing, psychology and sociology. The next question is: *Why not in the economic field?*

## Conclusions

This scoping review reveals a gap in the pet goods consumption field. We included all found studies in selected databases according to the certain rule of searching, only written in English. Based on our scope review pet goods consumption has not been analyzed so far in the way which allow to answer all questions from the economic perspective even all studies included in review presented in this paper were related to expenses on pet goods. Moreover, no advanced studies regarding pet goods consumption with references to economics topics as e.g., preferences and utility can be found either. This gap may occur due to the fact that pet goods consumption has not been treated in line with the other key economic issues such as households’ savings (microeconomics) or global growth (macroeconomics). Furthermore, the lack of standardized methodology of such analysis, well accepted in economic literature may have impacted this gap. But pets consumption have hardly been touched in any formal analysis, economics or otherwise.

However, the review has its limitations, the aim of the paper has been fulfilled. The results of scope review present new directions and findings that could be found in the literature concerning pet goods consumption, especially expenses on pet goods and pet owner economic choices.

Pets become a new family members and should be analyzed in the household economics field like analysis focusing on other household members. The modern lifestyle (putting of maternity, flexible working hours, and getting married latter) fosters household animals into society. Moreover, pets are set to grow in popularity due to COVID-19 (Oliva and Johnston 2021; Giansanti et al. 2022) and it also creates opportunities and hopefully more attention of economists to contribute with their theories to evaluate the impact of this type of the consumption.

## Data Availability

The articles analyzed during the current study are available from the corresponding author on reasonable request.

## Appendix A

“Pet goods consumption” Search terms for Electronic databases.

*Filter applied: published between 2000 and 2021, and document type is article*

TITLE-ABS-KEY ( "pet owner" OR "per related" OR "domestic pet*" OR "home pet*" OR "pet studies" OR "pet consumption" OR "pet* co-consumption" OR "pet* expenditure*" OR "keeping pet*" OR "spending on pet*" OR "pet spending" OR "pet* care cost*" OR "pet ownership cost") AND NOT TITLE-ABS-KEY-AUTH ( ( "polyethylene*" OR "petrol*" OR "plastic*" OR "polymeric*" OR "soil*" OR "oxygen*" OR "Physiologically equivalent temperatur" OR "nitrogen*")) AND KEY ( "consumption*" OR "expenditure*" OR "outgoing*" OR "outlay*" OR "expenses*" OR "outgoes*") AND NOT TITLE-ABS-KEY-AUTH ( "petrol*" OR "Physiologically equivalent temperatur" OR "plastic*" OR "polymeric*" OR "AAFP*" OR "AAHA*" OR "serotonine*" OR "tomography*" OR "SPECT*" OR "CNR*" OR "fMRI*" OR "tDCS*" OR "DBS*" OR "molecular*" OR "methyl*").

## Code availability

No specific software application or custom code was used in this study.

## References

[CR1] Ando A, Modigliani F (1963). The" life cycle" hypothesis of saving: Aggregate implications and tests. Am Econ Rev.

[CR2] APPA (2021) Pets by the numbers. Downloaded from: https://humanepro.org/page/pets-by-the-numbers [24.10.2021]

[CR3] Archer J (1997). Why do people love their pets?. Evol Hum Behav.

[CR4] Atkinson LZ, Cipriani A (2018). How to carry out a literature search for a systematic review: a practical guide. Bjpsych Advances.

[CR5] Becker G (1976). Economic approach to human behaviour.

[CR6] Brockman BK, Taylor VA, Brockman CM (2008). The price of unconditional love: Consumer decision making for high-dollar veterinary care. J Bus Res.

[CR7] Chaumet, A. C. S. G., Rossi, T. A., Murphy, L. A., & Nakamura, R. K. (2021). Evaluation of owners’ attitudes towards veterinary insurance in a specialty hospital. *Journal of Small Animal Practice.* 10.1111/jsap.13309 33587292

[CR8] Chee L (2017). Keeping cats, hoarding things: domestic situations in the public spaces of the Singaporean housing block. J Archit.

[CR9] Chen A, Hung KP, Peng N (2012). A cluster analysis examination of pet owners’ consumption values and behavior–segmenting owners strategically. J Target Meas Anal Mark.

[CR10] d’Ovidio D, Pirrone F (2018). A cross-sectional survey to evaluate the pet squirrel population and ownership profiles. Prev Vet Med.

[CR11] Duesenberry JS (1949) Income, saving, and the theory of consumer behavior

[CR12] Ellson T (2008). Can we live without a dog? Consumption life cycles in dog–owner relationship. J Bus Res.

[CR13] Frątczak-Rudnicka B (2015). Dwa miliardy na smyczy. Marketing w Praktyce.

[CR14] Friedman M (1957) A miscellany. In a theory of the consumption function (pp. 200–219). Princeton University Press

[CR15] Gates MC, Walker J, Zito S, Dale A (2019). Cross-sectional survey of pet ownership, veterinary service utilisation, and pet-related expenditures in New Zealand. N Z Vet J.

[CR16] Giansanti D, Siotto M, Parisi L, Aprile I (2022). Pet presence can reduce anxiety in the elderly: The Italian experience during COVID-19 lockdown assessed by an electronic survey. Int J Environ Res Public Health.

[CR17] Global marketing information data base (GMID) (2020) Euromonitor International. Downloaded from: https://www.portal.euromonitor.com/portal/ResultsList/Index, [01.11.2020].

[CR18] GMID (2022) World Market for Pet Care. Downloaded from: https://www-1portal-1euromonitor-1com-16a1ap8p203a4.han.sgh.waw.pl/portal/analysis/tab, [01.07.2022].

[CR19] Greenebaum J (2004). It’s a dog’s life: elevating status from pet to ‘fur baby’ at yappy hour. Soc Anim.

[CR20] Henderson S (2013) Spending on pets: “Tails” from the Consumer Expenditure Survey. Beyond the numbers, 2(16). Downloaded from: https://www.bls.gov/opub/btn/volume-2/pdf/spending-on-pets.pdf, [24.10.2020]

[CR21] Jyrinki H, Leipamaa-Leskinen H (2005) Pets as extended self in the context of pet food consumption. ACR European Advances

[CR22] Karen A (2003). Are pets a healthy pleasure? the influence of pets on blood. Curr Dir Psychol Sci.

[CR23] Keynes JM (1936). The general theory of employment, interest, and money: interest and money.

[CR24] Kim WH, Min KD, Cho SI, Cho S (2020). The Relationship between dog-related factors and owners' attitudes toward pets: an exploratory cross-sectional study in Korea. Front Veterin Sci.

[CR25] Kirk CP (2019). Dogs have masters, cats have staff: Consumers' psychological ownership and their economic valuation of pets. J Bus Res.

[CR26] Koppel K, Suwonsichon S, Chambers D, Chambers E (2018). Determination of intrinsic appearance properties that drive dry dog food acceptance by pet owners in Thailand. J Food Prod Market.

[CR27] Kylkilahti E, Syrjälä H, Autio J, Kuismin A, Autio M (2016). Understanding co-consumption between consumers and their pets. Int J Consum Stud.

[CR28] Lamour C, De La Robertie C (2016). Prescribed consumption and consumers' decision-making styles: a cross-cultural comparison between Europe and Asia. Int J Retail Distrib Manag.

[CR29] Leibenstein H. (1957). *Economic Backwardness and Economic Growth: Studies in the Theory of Economic Development*. Minnesota: Willey.

[CR30] Maharaj N, Kazanjian A, Borgen W (2018). Investing in human–animal bonds: What is the psychological return on such investment?. Loisir Et Société/society and Leisure.

[CR31] Medjo B, Atanaskovic-Markovic M, Nikolic D, Spasojevic-Dimitrijeva B, Ivanovski P, Djukic S (2013). Association between pet-keeping and asthma in school children. Pediatr Int.

[CR32] Morais Richard C (2004) “Dog Days,” Forbes.com, June 21.

[CR33] Mosteller J (2008). Animal-companion extremes and underlying consumer themes. J Bus Res.

[CR34] Office of National Statistics (2017) Family spending in the UK: financial year ending 2017. Downloaded from: https://www.ons.gov.uk/peoplepopulationandcommunity/personalandhouseholdfinances/expenditure/bulletins/familyspendingintheuk/financialyearending2017, [24.10.2020].

[CR35] Oliva JL, Johnston KL (2021). Puppy love in the time of Corona: Dog ownership protects against loneliness for those living alone during the COVID-19 lockdown. Int J Soc Psychiatry.

[CR36] Power ER (2017). Renting with pets: a pathway to housing insecurity?. Hous Stud.

[CR37] Pütz R, Poerting J (2020). Mensch-Tier-Verhältnisse in der Konsumgesellschaft | Human-animal relations in the consumer society. Berichte Zur Deutschen Landeskunde.

[CR38] Ridgway NM, Kukar-Kinney M, Monroe KB, Chamberlin E (2008). Does excessive buying for self relate to spending on pets?. J Bus Res.

[CR39] Schwarz PM, Troyer JL, Walker JB (2007). Animal house: Economics of pets and the household. BE J Econ Analysis Policy.

[CR40] Shirai T, Matsui T, Suzuki K, Chida K (2005). Effect of pet removal on pet allergic asthma. Chest.

[CR41] Syrjälä H (2016). Turning point of transformation: Consumer communities, identity projects and becoming a serious dog hobbyist. J Bus Res.

[CR42] Tesfom G, Birch N (2010). Do they buy for their dogs the way they buy for themselves?. Psychol Mark.

[CR43] The Harris Poll (2012) Pets Aren’t Just Animals; They are Members of the Family. Downloaded from: https://theharrispoll.com/new-york-n-y-september-13-2012-Americans-have-always-had-interesting-relationships-with-their-pets-whether-those-pets-are-cats-dogs-parakeets-or-something-else-entirely-the-pet-industry-i/, [06.09.2020].

[CR44] Vänskä A (2014). New kids on the mall: Babyfied dogs as fashionable co-consumers. Young Consumers: Insight and Ideas for Responsible Marketers.

[CR45] Vänskä A (2016) 'Cause I wuv you!'Pet dog fashion and emotional consumption. ephemera: theory & politics in organization, 16(4)

[CR500] Varian HR (2019) Mikroekonomia. Kurs średni-ujęcie nowoczesne. PWN, Warszawa

[CR46] Veblen T (1899) 1994 The theory of the leisure class: an economic study of institutions

[CR47] Williams A, Williams B, Hansen CR, Coble KH (2020). The impact of pet health insurance on dog owners’ spending for veterinary services. Animals.

[CR48] Wolf CA, Lloyd JW, Black JR (2008). An examination of US consumer pet-related and veterinary service expenditures, 1980–2005. J Am Vet Med Assoc.

[CR49] Zasloff RL, Kidd AH (1994). Loneliness and pet ownership among single women. Psychol Rep.

